# High-quality draft genome sequence of the *Thermus amyloliquefaciens* type strain YIM 77409^T^ with an incomplete denitrification pathway

**DOI:** 10.1186/s40793-016-0140-3

**Published:** 2016-02-27

**Authors:** En-Min Zhou, Senthil K. Murugapiran, Chrisabelle C. Mefferd, Lan Liu, Wen-Dong Xian, Yi-Rui Yin, Hong Ming, Tian-Tian Yu, Marcel Huntemann, Alicia Clum, Manoj Pillay, Krishnaveni Palaniappan, Neha Varghese, Natalia Mikhailova, Dimitrios Stamatis, T. B. K. Reddy, Chew Yee Ngan, Chris Daum, Nicole Shapiro, Victor Markowitz, Natalia Ivanova, Alexander Spunde, Nikos Kyrpides, Tanja Woyke, Wen-Jun Li, Brian P. Hedlund

**Affiliations:** Yunnan Institute of Microbiology, Yunnan University, Kunming, 650091 People’s Republic of China; School of Life Sciences, University of Nevada Las Vegas, Las Vegas, NV USA; State Key Laboratory of Biocontrol and Guangdong Provincial Key Laboratory of Plant Resources, College of Ecology and Evolution, Sun Yat-Sen University, Guangzhou, 510275 People’s Republic of China; Department of Energy Joint Genome Institute, Walnut Creek, CA USA; Nevada Institute of Personalized Medicine, University of Nevada Las Vegas, Las Vegas, NV USA

**Keywords:** *Thermus*, *Thermus amyloliquefaciens*, Thermophiles, Hot springs, Denitrification

## Abstract

*Thermus amyloliquefaciens* type strain YIM 77409^T^ is a thermophilic, Gram-negative, non-motile and rod-shaped bacterium isolated from Niujie Hot Spring in Eryuan County, Yunnan Province, southwest China. In the present study we describe the features of strain YIM 77409^T^ together with its genome sequence and annotation. The genome is 2,160,855 bp long and consists of 6 scaffolds with 67.4 % average GC content. A total of 2,313 genes were predicted, comprising 2,257 protein-coding and 56 RNA genes. The genome is predicted to encode a complete glycolysis, pentose phosphate pathway, and tricarboxylic acid cycle. Additionally, a large number of transporters and enzymes for heterotrophy highlight the broad heterotrophic lifestyle of this organism. A denitrification gene cluster included genes predicted to encode enzymes for the sequential reduction of nitrate to nitrous oxide, consistent with the incomplete denitrification phenotype of this strain.

## Introduction

*Thermus* species have been isolated from both natural and man-made thermal environments such as hot springs, hot domestic water, deep mines, composting systems, and sewage sludge [1–5]. The genus has attracted considerable attention as a source of thermostable enzymes, which have important biotechnological applications [6], and as a model organism to study the mechanisms involved in bacterial adaptation to extreme environments [7]. Members of the genus *Thermus* were formerly considered to be strictly aerobic, based on the characteristics of the type species *Thermus aquaticus* [2]. However, many studies have shown that *Thermus* strains also can grow as facultative anaerobes using nitrogen oxides, sulfur, or metals as terminal electron acceptors under oxygen-deprived conditions [8–10]. Cava et al. [11] demonstrated that different *T. thermophilus* strains can grow anaerobically by reducing nitrate to nitrite or by reducing nitrite to a gaseous nitrogen product.

The nitrogen biogeochemical cycle has been investigated in a few geothermal systems [12], including Great Boiling Spring, a ~80 °C hot spring in the U.S. Great Basin [13–15]. Studies in GBS revealed a high flux of nitrous oxide, particularly in the ~80 °C source pool, suggesting the importance of incomplete denitrifiers in high-temperature environments. A subsequent cultivation and physiological study of heterotrophic denitrifiers suggested a significant role of *T. oshimai* and *T. thermophilus* in denitrification in this hot spring [16]. A following study of the whole genomes of one strain from each species, *T. oshimai* JL-2 and *T. thermophilus* JL-18, revealed that they have genes encoding the sequential reduction of nitrate to nitrous oxide but lack genes encoding the nitrous oxide reductase, and explains their incomplete denitrification phenotype [17].

*Thermus amyloliquefaciens* strain YIM 77409^T^ was isolated in the course of an investigation of the culturable thermophiles that inhabit geothermal springs in Yunnan Province, southwest China [18]. Strain YIM 77409^T^ was cultured from a sediment sample collected from Niujie Hot Spring using the serial dilution technique on T5 agar. This organism was able to grow anaerobically using nitrate as a terminal electron acceptor, and may potentially impact the nitrogen biogeochemical cycle. Here we describe a summary classification and a set of the features of *Thermus amyloliquefaciens* type strain YIM 77409^T^, together with the genome sequence description and annotation. This work may help to better understand the physiological characters as well as the ecological role of this organism in hot spring ecosystems.

## Organism information

### Classification and features

**Fig. 1:**
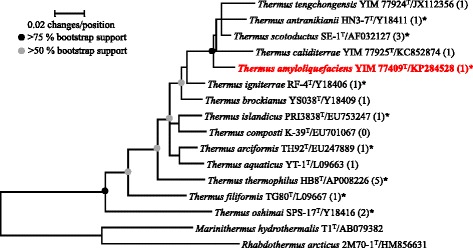
Maximum-likelihood phylogenetic tree of the genus *Thermus* to highlight the position of *Thermus amyloliquefaciens* strain YIM 77409^T^. The tree was reconstructed based on 1374 aligned positions that remained after the application of the Lane mask to the 16S rRNA gene sequences using MEGA 5.0 [54]. Complete deletion of gaps and missing data and Kimura’s two-parameter model was applied. Bootstrap analysis was based on 1000 resamplings. Nodes supported in >75 % (black circles) or >50 % (grey circles) of bootstrap pseudoreplicates (1000 resamplings) for both maximum-likelihood and neighbor-joining methods are indicated. Bar, 0.02 changes per nucleotide. The number of genomes available for each species is included in parentheses (see Table 5) and the asterisk indicates that the genome of the type strain is available. The 16S rRNA gene sequences from *Marinithermus hydrothermalis* T1^T^/AB079382 and *Rhabdothermus arcticus* 2 M70-1^T^/HM856631 were used as outgroups

A taxonomic study using a polyphasic approach placed strain YIM 77409^T^ in the genus *Thermus* within the family *Thermaceae* of the phylum *Deinococcus-Thermus* and resulted in the description of a novel species, *Thermus amyloliquefaciens**,* according to its ability to digest starch [18]. The highest 16S rRNA gene sequence pairwise similarities for strain YIM 77409^T^ were found with the type strain of *T. scotoductus* SE-1^T^ (97.6 %), *T. antranikianii* HN3-7^T^ (96.6 %), *T. caliditerrae*YIM 77925^T^ (96.5 %), and *T. tengchongensis*YIM 77924^T^ (96.1 %) using EzTaxon-e [19]. The sequence similarities were less than 96.0 % with all other species. Phylogenetic analyses based on the 16S rRNA gene sequences show that YIM 77409^T^ together with *T. caliditerrae*, *T. scotoductus*, *T. antranikianii*, and *T. tengchongensis* constitute a distinct monophyletic group within the genus *Thermus* (Fig. 1). The DNA-DNA hybridization value between strains YIM 77409^T^ and *T. scotoductus* SE-1^T^ was 30.6 ± 1.6 % [18], which was lower than the threshold value (70 %) for the recognition of microbial species [20]. Similarly, the average nucleotide identity (ANI) score between the two strains based on genome-wide comparisons was 86.6  %, according to the algorithm proposed by Goris et al. [21], which is lower than the ANI threshold range (95–96 %) for species demarcation [22]. Those results indicate that strain YIM 77409^T^ represents a distinct genospecies in the genus *Thermus* [18].

**Fig. 2:**
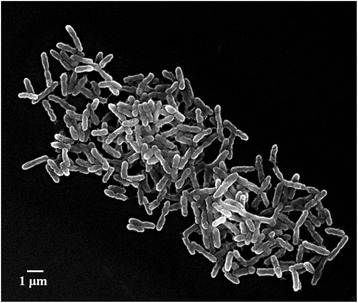
Scanning electron microscopy image of *Thermus amyloliquefaciens* strain YIM 77409^T^ grown in *Thermus* medium broth at 65 °C for 24 h

Strain YIM 77409^T^ is Gram-negative, facultatively anaerobic, non-motile, and rod shaped (Fig. 2). Cells are 0.4–0.6 μm wide and 1.5–4.5 μm long. Colonies grown on an R2A, T5, and *Thermus* agar plates for 2 days are yellow and circular. The strain degrades starch and is positive for nitrate reduction. The predominant menaquinone is MK-8. Major fatty acids (>10 %) are iso-C15:0 and iso-C17:0. The polar lipids consist of aminophospholipid, one unidentified phospholipid, and two unidentified glycolipids. Minimum Information about the Genome Sequence [23] of type strain YIM 77409^T^ is provided in Table 1.

**Table 1:** Classification and general features of *Thermus amyloliquefaciens* strain YIM 77409^T^ [23]

MIGS ID	Property	Term	Evidence code^a^
	Classification	Domain *Bacteria*	TAS [45]
		Phylum *Deinococcus-Thermus*	TAS [46]
		Class *Deinococci*	TAS [47, 48]
		Order *Thermales*	TAS [48, 49]
		Family *Thermaceae*	TAS [48, 50]
		Genus *Thermus*	TAS [2, 51, 52]
		Species *Thermus amyloliquefaciens*	TAS [18]
		Type strain: YIM 77409^T^	TAS [18]
	Gram stain	Negative	TAS [18]
	Cell shape	Rod	TAS [18]
	Motility	Non-motile	TAS [18]
	Sporulation	Nonsporulating	TAS [18]
	Temperature range	50–70 °C	TAS [18]
	Optimum temperature	60–65 °C	TAS [18]
	pH range; Optimum	6.0–8.0; 7.0	TAS [18]
	Carbon source	Glucose, sucrose, glycerol, maltose, raffinose, trehalose, rhamnose, inositol, xylitol, mannitol, sodium malate, mannose and *L*-arabinose	TAS [18]
MIGS-6	Habitat	Terrestrial hot springs	TAS [18]
MIGS-6.3	Salinity	Not reported	
MIGS-22	Oxygen requirement	Facultatively anaerobic	TAS [18]
MIGS-15	Biotic relationship	Free-living	TAS [18]
MIGS-14	Pathogenicity	Non-pathogen	NAS
MIGS-4	Geographic location	Niujie hot spring in Eryuan County, Yunnan Province, southwest China	TAS [18]
MIGS-5	Sample collection	2010	NAS
MIGS-4.1	Latitude	N 26°15'01. 4"	NAS
MIGS-4.2	Longitude	E 99°59'22. 3"	NAS
MIGS-4.4	Altitude	2060 m	NAS

*IDA* Inferred from Direct Assay, *TAS* Traceable Author Statement (i.e., a direct report exists in the literature), *NAS* Non-traceable Author Statement (i.e., not directly observed for the living, isolated sample, but based on a generally accepted property for the species, or anecdotal evidence). These evidence codes are from the Gene Ontology project [53]

^a^ Evidence codes

## Genome sequencing information

### Genome project history

*T. amyloliquefaciens* strain YIM 77409^T^ was selected for whole genome sequencing based on its phylogenetic position, denitrifying phenotype, and also for its biotechnological potential. Comparison of the genome of this organism to that of other sequenced *Thermus* species may provide insights into the molecular basis of the denitrification process in this genus. The genome project for strain YIM 77409^T^ was deposited in the Genomes OnLine Database [24] and the complete sequences were deposited in GenBank. Sequencing, finishing, and annotation were performed by the Department of Energy Joint Genome Institute (Walnut Creek, CA, USA) using state of the art sequencing technology [25]. A summary of the project information associated with MIGS version 2.0 compliance [23] is shown in Table 2.

**Table 2:** Project information

MIGS ID	Property	Term
MIGS 31	Finishing quality	Permanent Draft
MIGS-28	Libraries used	PacBio 10 kb
MIGS 29	Sequencing platforms	PacBio RS
MIGS 31.2	Fold coverage	384.9X PacBio
MIGS 30	Assemblers	HGAP version 2.1.1
MIGS 32	Gene calling method	Prodigal 2.5; GenePRIMP
	Locus Tag	BS74
	Genbank ID	JQMV00000000
	GenBank Date of Release	August 28, 2014
	Database: IMG	2579778517
	GOLD ID	Gp0050852
	BIOPROJECT	PRJNA234787
MIGS 13	Source Material Identifier	YIM 77409^T^
	Project relevance	Biotechnological

### Growth conditions and genomic DNA preparation

*T. amyloliquefaciens* type strain YIM 77409^T^ was grown aerobically in *Thermus* medium at 65 °C for 2 days [18] and DNA was isolated from 0.5–1.0 g of cell pellet using the Joint Genome Institute CTAB bacterial genomic DNA isolation protocol [26].

### Genome sequencing and assembly

The draft genome of *T. amyloliquefaciens* type strain YIM 77409^T^ was generated at the DOE JGI using Pacific Biosciences sequencing technology [27]. A PacBio SMRTbell™ library was constructed and sequenced on the PacBio RS platform using three SMRT cells, which generated 264,235 filtered subreads totaling 751.5 Mbp with an N50 contig length of 2,065,958 bp. All general aspects of library construction and sequencing can be found at the JGI website. All raw reads were assembled using HGAP version 2.1.1 [28]. The final draft assembly produced 6 contigs in 6 scaffolds, totaling 2.16 Mbp in size. The input read coverage was 384.9 × .

### Genome annotation

Genes were identified using Prodigal [29] as part of the JGI microbial annotation pipeline [30], followed by a round of manual curation using the JGI GenePRIMP pipeline [31]. The predicted coding sequences were translated and used to search against the Integrated Microbial Genomes non-redundant database, UniProt, TIGRfam, Pfam, PRIAM, KEGG, COG, and InterPro databases. These data sources were combined to assert a product description for each predicted protein. The rRNA genes are predicted using hmmsearch tool from the package HMMER 3.0 [32] and a set of in-house curated HMMs derived from an alignment of full-length rRNA genes selected from IMG isolate genomes; tRNA genes were found using tRNAscan-SE 1.3.1 [33]; other non-coding RNAs and regulatory RNA features were found by searching the genome for the corresponding Rfam profiles using INFERNAL 1.0.2 package [34]. Additional gene prediction analysis and manual functional annotation was performed using the Integrated Microbial Genomes Expert Review platform developed by the JGI [35]. The analysis of the genome presented here and the annotations are for the version available through IMG (2579778517).

## Genome properties

The *T. amyloliquefaciens*YIM 77409^T^ high quality draft genome is 2,160,855 bp long with a 67.4 % G + C content. The genomes comprise 2,257 protein-coding genes and 56 RNA genes. The coding regions accounted for 94 % of the whole genome and 1,839 genes were assigned to a putative function with the remaining annotated as hypothetical proteins. A total of 1,558 genes (67.4 %) were assigned to COGs. The properties and the statistics of the genome are presented in Table 3. The distribution of genes into COG functional categories is presented in Table 4.

**Table 3:** Genome statistics

Attribute	Value	% of Total^a^
Genome size (bp)	2,160,855	100.0
DNA coding (bp)	2,031,100	94.0
DNA G + C (bp)	1,457,281	67.4
DNA scaffolds	6	100.0
Total genes	2,313	100.0
Protein coding genes	2,257	97.6
RNA genes	56	2.4
Pseudo genes^b^	74	3.2
Genes in internal clusters	1,932	83.5
Genes with function prediction	1,839	79.5
Genes assigned to COGs	1,558	67.4
Genes with Pfam domains	1,842	79.6
Genes with signal peptides	110	4.8
Genes with transmembrane helices	439	19.0
CRISPR repeats	5	

^a^The total is based on either the size of the genome in base pairs or the total number of protein coding genes in the annotated genome

^b^Pseudogenes may also be counted as protein coding or RNA genes, so is not additive under total gene count

**Table 4:** Number of genes associated with general COG functional categories

Code	Value	%age	Description
J	179	10.4	Translation, ribosomal structure and biogenesis
A	4	0.2	RNA processing and modification
K	76	4.4	Transcription
L	63	3.7	Replication, recombination and repair
B	2	0.1	Chromatin structure and dynamics
D	22	1.3	Cell cycle control, Cell division, chromosome partitioning
V	35	2.0	Defense mechanisms
T	66	3.8	Signal transduction mechanisms
M	77	4.5	Cell wall/membrane biogenesis
N	16	0.9	Cell motility
U	14	0.8	Intracellular trafficking and secretion
O	90	5.2	Posttranslational modification, protein turnover, chaperones
C	131	7.6	Energy production and conversion
G	105	6.1	Carbohydrate transport and metabolism
E	183	10.6	Amino acid transport and metabolism
F	75	4.4	Nucleotide transport and metabolism
H	121	7.0	Coenzyme transport and metabolism
I	91	5.3	Lipid transport and metabolism
P	82	4.8	Inorganic ion transport and metabolism
Q	40	2.3	Secondary metabolites biosynthesis, transport and catabolism
R	170	9.9	General function prediction only
S	68	4.0	Function unknown
-	755	32.6	Not in COGs

The total is based on the total number of protein coding genes in the genome

## Insights from the genome sequence

### Comparisons with other *Thermus* spp. genomes

Twenty-two *Thermus* genomes from 12 different species have been sequenced, including *T. amyloliquefaciens* type strain YIM 77409^T^, and 7 of them have finished genome sequences. The phylogenetic coverage of these genomes is shown in Fig. 1 and their basic properties are summarized in Table 5. The *Thermus* genomes range in size from 2.04 Mb (*Thermus* sp. RLM) to 2.56 Mb (*T. tengchongensis*YIM 77401); GC contents vary from 64.8 % (*T. scotoductus*DSM 8553^T^) to 69.5 % (*T. thermophilus* HB8^T^), predicted gene number range from 2,043 (*T.* sp. RLM) to 2,789 (*T. brockianus*). The genome size (2.16 Mb) and GC contents (67.4 %) of strain YIM 77409^T^ are around the average value, but the gene number of this strain is lower than the average, possibly indicating gene loss through genomic streamlining in this species. In addition, the percentage of protein-coding genes with functional prediction (79.5 %) is higher than the average, whereas the percentage of protein-coding genes with COGs (67.4 %) is similar to the average of the genus *Thermus*.

**Table 5:** Comparison of basic genome features of *Thermus* strains

Genome Name	Status	Genome Size (Mb)	GC Content (%)	Gene Count	No. of protein coding genes with function prediction	Percentage (%)	No. of protein coding genes with COGs	Percentage (%)	IMG Genome ID
*T. amyloliquefaciens* YIM 77409^T^	Draft	2.16	67.4	2313	1839	79.5	1558	67.4	2579778517
*T. scotoductus* SA-01	Finished	2.36	64.9	2514	1878	74.7	1704	67.8	649633105
*T. scotoductus* KI2	Draft	2.48	65.5	2643	2159	81.7	1808	68.4	2574179778
*T. scotoductus* DSM 8553^T^	Draft	2.07	64.8	2305	1816	78.8	1484	64.4	2518645614
*T. antranikianii* DSM 12462^T^	Draft	2.17	64.8	2321	1939	83.5	1654	71.3	2522572193
*T. caliditerrae* YIM 77777^T^	Draft	2.22	67.2	2327	1901	81.7	1646	70.7	2582581225
*T. tengchongensis* YIM 77401	Draft	2.56	66.4	2750	2158	78.5	1818	66.1	2574179781
*T. arciformis* CGMCC 1.6992^T^	Draft	2.44	68.7	2672	2052	76.8	1704	63.8	2617270932
*T. thermophilus* HB8^T^	Finished	2.12	69.5	2302	1498	65.1	1550	67.3	637000323
*T. thermophilus* JL-18	Finished	2.31	69.0	2508	1984	79.1	1717	68.5	2508501108
*T. thermophilus* SG0.5JP17-16	Finished	2.30	68.7	2488	2024	81.4	1700	68.3	2505679077
*T. thermophilus* HB27	Finished	2.13	69.4	2273	1517	66.7	1562	68.7	637000322
*T. thermophilus* ATCC 33923	Draft	2.15	69.4	2366	1928	81.5	1603	67.8	2554235155
*T. islandicus* DSM 21543^T^	Draft	2.26	68.4	2470	1965	79.6	1654	67.0	2524614852
*T. oshimai* JL-2	Finished	2.40	68.6	2548	2018	79.2	1735	68.1	2508501045
*T. oshimai* DSM 12092^T^	Draft	2.26	68.7	2409	1960	81.4	1700	70.6	2515154080
*T. igniterrae* ATCC 700962^T^	Draft	2.23	68.8	2379	1962	82.5	1661	69.8	2515154172
*T. aquaticus* Y51MC23	Draft	2.34	68.1	2595	1740	67.1	1530	59.0	645058872
*T. brockianus*	Draft	2.48	66.8	2789	2004	71.9	1709	61.3	2502171156
*T.* sp. CCB_US3_UF1	Finished	2.26	68.6	2333	1935	82.9	1655	70.9	2511231187
*T.* sp. RLM	Draft	2.04	68.3	2043	1636	80.1	1326	64.9	2513237279
*T.* sp. NMX2.A1	Draft	2.29	65.3	2522	1954	77.5	1666	66.1	2514885041

### Profiles of metabolic network and pathway

The *T. amyloliquefaciens*YIM 77409^T^ genome encodes genes for complete glycolysis, gluconeogenesis, tricarboxylic acid cycle, pyruvate dehydrogenase, and pentose phosphate pathway. Twenty ABC transporters were identified in the YIM 77409^T^ genome, including amino acid, oligopeptide/dipeptide, *N*-acetyl-*D*-glucosamine, maltose, nucleoside, sugar, phosphonate, phosphate, thiamin, cation, and ammonium transporters as well as other permeases. The genome also encodes glucosidases, glycosidases, proteases, and peptidases. The finding of three genes probably coding for esterase (BS74_RS04020, BS74_RS04625, BS74_RS10315) and one gene probably coding for amylopullulanase (BS74_RS00620) are consistent with the observed lipase and amylase activities observed in strain YIM 77409^T^. A number of genes assigned to a classical electron transport chain have been identified in the strain YIM 77409^T^ genome. Respiratory complex I NADH quinone oxidoreductases consists of NADH quinone oxidoreductase chains A-N (BS74_RS03070-BS74_RS03135), NADH quinone oxidoreductase subunit 15 (BS74_RS02790), and two quinone oxidoreductases (BS74_RS00610, BS74_RS06600). Complex II consists of succinate dehydrogenase (cytochrome *b*_556_ subunit SdhC (BS74_RS07950), SdhA (BS74_RS07940), SdhB (BS74_RS07935), and SdhD (BS74_RS07945). A four-subunit cytochrome *bc*_1_ complex found in *T. thermophilus* was also identified in strain YIM 77409^T^ (BS74_RS10415-BS74_RS10430) [36, 37]. The terminal cytochrome oxidase is encoded by four cytochrome c oxidase genes *ctaC1* (BS74_RS00820), *caaA* (BS74_RS00825), *ctaD2* (BS74_RS04775), and *ctaC2* (BS74_RS04780). Other cytochrome c oxidase genes observed in *T. scotoductus* SA-01, *ctaH*, *ctaE1*, *ctaE2*, *ctaD1*, and *coxM* (TSC_C00960-TSC_C01000), were not found in the YIM 77409^T^ genome.

### Genes involved in denitrification

**Fig. 3:**
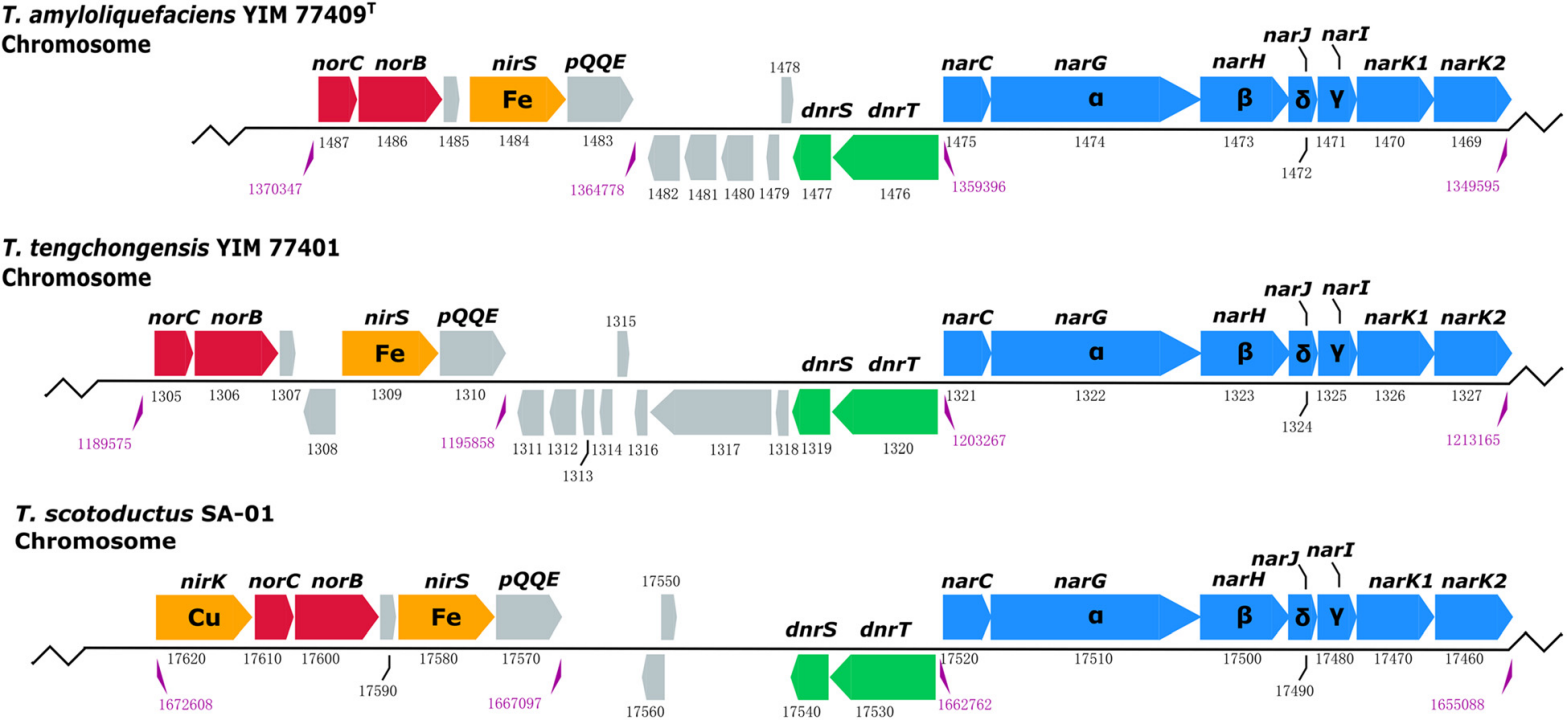
Molecular organization of identified *nar* operon and neighboring genes involved in denitrification located on the chromosome of *T. amyloliquefaciens* YIM 77409^T^, *T. tengchongensis* YIM 77401, and *T. scotoductus* SA-01. Fe: heme protein-containing nitrite reductase, Cu: copper-containing nitrite reductase. Numbers below the genes indicate the provisional ORF numbers in *T. amyloliquefaciens* YIM 77409^T^ and *T. tengchongensis* YIM 77401, the locations in the chromosome are indicated below. *nar*: nitrate reductase gene; *nir*: nitrite reductase gene; *nor*: nitric oxide reductase gene; *dnr*: denitrification regulator gene [43, 55, 56]. This figure is modified from Murugapiran et al. [17]

Denitrification is a respiratory process to reduce nitrate or nitrite stepwise to nitrogen gas (NO_3_^−^ → NO_2_^−^ → NO → N_2_O → N_2_), and plays a major role in converting bioavailable nitrogen to recalcitrant dinitrogen gas [38]. Denitrification normally occurs under oxygen-limiting conditions, and is catalyzed by four types of nitrogen oxide reductases in sequence: nitrate reductase (Nar or Nap), nitrite reductase (Nir), nitric oxide reductase (Nor), and nitrous oxide reductase (Nos) [39, 40]. Previous studies have demonstrated that some *Thermus* species have incomplete denitrification phenotypes terminating with the production of nitrite or nitrous oxide [16, 41]. This incomplete denitrification is partly encoded by a conjugative element (nitrate conjugative element, NCE) that can be transferred among strains [42]. The NCE is composed of two main operons, *nar* and *nrc*, and the transcription factors DnrS and DnrT, which are required for their expression under anaerobic conditions when nitrate is present [43, 44]. The periplasmic nitrate reductase subunits NapB and NapC were not found in the genome of *T. amyloliquefaciens*YIM 77409^T^, consistent with the use of the Nar system in the *Thermales*. Figure 3 shows the organization of the *nar* operon and neighboring genes involved in denitrification in *T. amyloliquefaciens*YIM 77409^T^, *T. tengchongensis*YIM 77401, and *T. scotoductus* SA-01. They are located on the chromosome in strains YIM 77409^T^ and YIM 77401, as in *T. scotoductus* SA-01. However, these gene clusters are located on megaplasmids in *T. thermophilus* and *T. oshimai* strains [17]. The *nar* operons show a high degree of synteny and consist of *narCGHJIKT* encoding the associated periplasmic cytochrome NarC, the membrane-bound nitrate reductase (NarGHI), the dedicated chaperone NarJ, the nitrate/proton symporter (NarK1), which might also function in nitrite extrusion in *T. thermophilus* HB8^T^, and the nitrate/nitrite antiporter (NarK2). Regulatory protein A and a denitrification regulator gene operon *dnrST* are adjacent to the *nar* operons. Strain YIM 77409^T^ contains a putative *nirS*, which encodes the isofunctional tetraheme cytochrome *cd*1-containing nitrite reductase. The *nirK*, encoding a Cu-containing nitrite reductase in *T. scotoductus* SA-01, is absent in strain YIM 77409^T^ and YIM 77401. Genes encoding conserved hypothetical proteins, coenzyme PQQ synthesis protein (PqqE), and nitric oxide reductase subunit *b* (NorB) and *c* (NorC) were also presented in the YIM 77409^T^ genome. Genes encoding the periplasmic multicopper enzyme nitrous oxide reductase (Nos), which catalyzes the last step of the denitrification (N_2_O → N_2_), were not observed in the YIM 77409^T^ genome or in any *Thermus* spp. genomes. Physiological experiments with nitrate as the sole terminal electron acceptor also confirm that strain YIM 77409^T^ can convert nitrate to nitrous oxide under anaerobic conditions, but not to nitrogen gas.

## Conclusions

The genus *Thermus* is the archetypal thermophilic bacterium and has been isolated from both natural and man-made thermal environments. Members of this genus are of significance as a source of thermophilic enzymes of great biotechnological interest and as an excellent laboratory models to study the molecular basis of thermal stability. Here, we report the annotation of a high quality draft genome sequence of *Thermus amyloliquefaciens*YIM 77409^T^. Analysis of the genome revealed that strain YIM 77409^T^ encodes enzymes involved in complete glycolysis, pentose phosphate pathway, tricarboxylic acid cycle, pyruvate dehydrogenase, and pentose phosphate pathway. The genome sequence of strain YIM 77409^T^ provides insights to better understand the molecular mechanisms of the incomplete denitrification phenotype and the ecological roles that *Thermus* species play in nitrogen cycling. Combined analysis of this genome and other *Thermus* genomes also provides important insights into the evolution and ecology of this group and the role it may play in the high-temperature nitrogen biogeochemical cycle.

## Abbreviations

GBS: Great Boiling Spring
CTAB: cetyl trimethyl ammonium bromide
PacBio: Pacific Biosciences
ANI: average nucleotide identity
NCE: nitrate conjugative element
